# Field‐Programmable Mobile Magneto‐Photonic Metaparticles for Active Light Manipulation and Steering

**DOI:** 10.1002/adma.74028

**Published:** 2026-07-15

**Authors:** Seung Yeol Lee, Yujie Luo, Ognjen Ilic

**Affiliations:** ^1^ Department of Mechanical Engineering University of Minnesota Minneapolis USA

**Keywords:** active beam steering, functional microparticles, magnetic actuation, mobile optical elements, nanoimprint lithography, optical metasurfaces

## Abstract

Controlling the flow of light within complex and dynamic environments is essential for a wide range of applications, from deep‐tissue imaging and optogenetics to precision phototherapy. Typically, such light flows are controlled using external optical systems requiring line‐of‐sight access or by embedded nanoparticle scatterers with limited directional control, underscoring the need for mobile photonic agents capable of actively delivering and steering light within complex media. Here, we present magneto‐photonic metaparticles: mobile, magnetically actuated microstructures that integrate a magnetic core with a nanoimprinted photonic surface. This hybrid design merges the reconfigurability of photonic metasurfaces with the mobility of magnetic actuation, enabling programmable translation, rotation, and real time beam steering in aqueous media. In a proof‐of‐concept demonstration, we realize polymeric metaparticles with an embedded magnetic core and a nanoimprinted surface that exhibit controlled locomotion and active, magnetically programmable beam steering. Our design approach further points to more sophisticated metaparticle designs with high‐efficiency, polarization‐insensitive light steering, compatible with a scalable, single‐step nanoimprint process. The mobile magneto‐photonic metaparticle platform combines metasurface‐level optical control with magnetic mobility, offering a versatile and scalable approach for active photonic control in complex environments.

## Graphical abstract

We report magneto‐photonic metaparticles that integrate magnetic actuation with nanoimprinted metagratings, enabling mobile and programmable beam steering in aqueous environments. This platform achieves real time, directional light control beyond static or line‐of‐sight systems, while remaining compatible with scalable fabrication, providing a practical route toward active photonic manipulation.

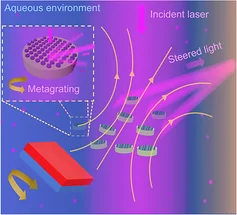

## 1 Introduction

The ability to deliver light of desired properties, such as wavelength, intensity, and polarization, to precise locations in complex media is critical for many applications across biology and biomedicine, from neural stimulation [1, 2] and imaging [3] to drug delivery [4] and thermotherapy [5]. In biological and aqueous environments, this task becomes especially challenging because targets are rarely stationary: cells drift with fluid flow [6, 7], and tissues or organs exhibit micromotion [8, 9]. Conventional optical systems and probes [10], which rely on external beam steering, are poorly suited for such dynamic conditions: external illumination systems are sensitive to alignment as targets move or deform, and they require line‐of‐sight access to be effective. These limitations underscore the need for embedded light‐delivery agents: miniaturized, mobile platforms capable of controlling the flow of light within the environment of interest. Such in situ control would enable real time beam steering, allowing precise delivery and collection of light for applications ranging from optical stimulation [11] to imaging [12] and diagnostics. Achieving this, however, demands an active photonic system that combines compactness, responsiveness, and engineered optical functionality, all within microscale dimensions.

One approach toward miniaturized light delivery involves using nanoparticles as mobile optical agents. Metal and dielectric nanoparticles [4, 13, 14, 15, 16] can scatter light within aqueous environments, and their optical response can, in principle, be harnessed for imaging or therapeutic purposes. Isotropic particles [17], such as spherical nanoparticles, offer limited means of controlling the directionality of scattered light due to their symmetric geometry. Introducing anisotropy through nanorods [18], dimers [19], Janus particles [20, 21], or core‐shell particles [22] can provide partial control over polarization or directional scattering; however, the orientation of these particles in a fluidic environment is easily randomized by Brownian motion and hydrodynamic forces, making stable directional control challenging. Furthermore, the subwavelength scale of such particles fundamentally limits the optical response they can support, making it impossible to implement advanced photonic functionality, such as beam steering, in such a small form.

By contrast, the level of photonic functionality unattainable in nanoparticles has been realized in metasurfaces—engineered structures that have attracted significant recent attention due to their unprecedented ability to shape light flows. These subwavelength‐engineered surfaces [23, 24, 25] have transformed optics and photonics, enabling precise manipulation of phase, amplitude, and polarization for applications in optical communication [26, 27, 28], imaging [29, 30], and optical sensing [31, 32]. However, despite their versatility, metasurfaces are typically realized as immobile and substrate‐bound devices. Even when tuned electrically [33], thermally [34, 35], or mechanically [36, 37], they remain substrate‐bound, lacking the ability to operate freely within three‐dimensional (3D) or aqueous environments. This immobility fundamentally restricts their use in configurations demanding in situ adaptability or mobility. Related advances at the interface of light and microscale actuation have shown that optical fields can drive, deform, or organize microsystems, enabling photoresponsive microrobots for biomimetic swimming [38] and collective locomotion applications [39]. Recent work has also incorporated magnetically and optically driven microrobots for beam shaping [40] and propulsion applications [41]. However, these systems couple optical response to the mechanical mechanism through material deformation, photothermal effects, or optical momentum transfer. Additionally, such platforms may often require serial direct‐write lithography, which limits scalable fabrication. These related approaches are compared in Table S1.

**FIGURE 1:**
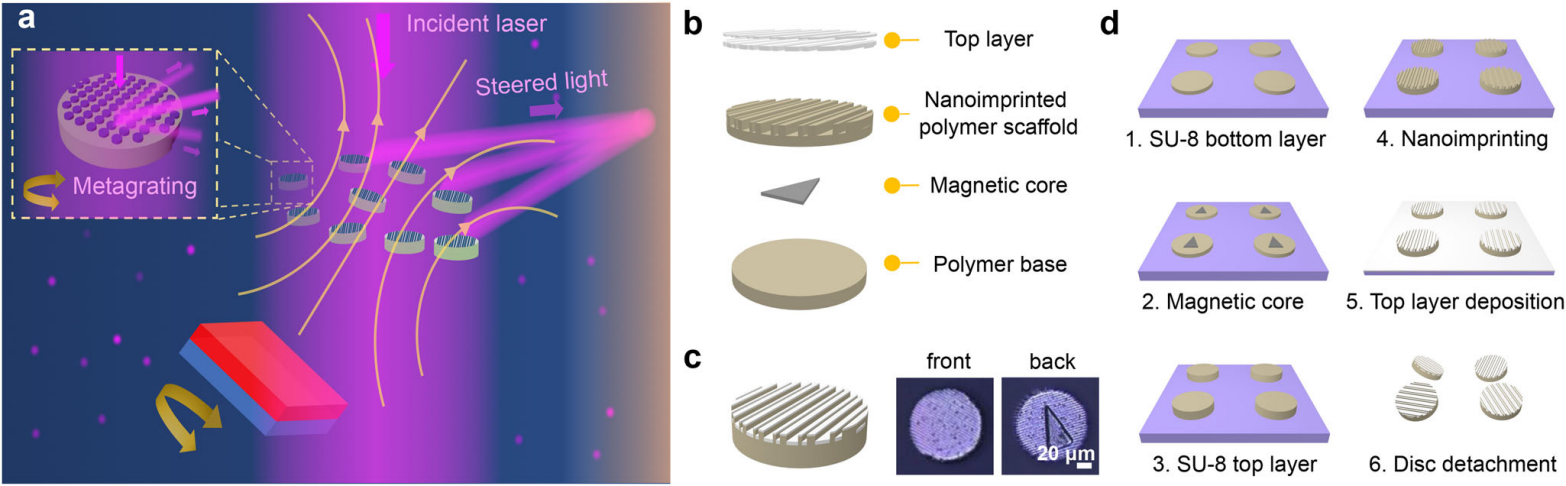
Concept, structure, and fabrication of magneto‐photonic metaparticles. (a) Schematic illustration showing a metaparticle with a photonic surface pattern and an embedded magnetic core. This hybrid design merges the reconfigurability of photonic metasurfaces with the mobility of magnetic actuation, enabling programmable collective motion and beam steering. (b) Structural anatomy, (c) illustration and optical microscope image, and (d) fabrication process of an integrated magneto‐photonic metaparticle.

Here, we present a new approach that combines the advanced photonic control of beam steering with the mobility of scalable particle‐based systems, as shown in Figure 1a. We introduce magneto‐photonic metaparticles: embeddable, magnetically actuated microstructures that integrate a magnetic core with a nanoimprinted photonic surface. This configuration enables precise motion and orientation control through external magnetic fields while maintaining full, independently designed, photonic functionality for targeted light delivery. By transitioning from an immobile, macroscale and substrate‐bound metasurface to a mobile, particle‐based architecture, this approach merges the versatility of metasurface optics with the spatial freedom and non‐line‐of‐sight control of magnetic actuation. Together, these features yield a scalable, field‐programmable photonic platform capable of real time beam steering in dynamic environments, as conceptually illustrated in Figure 1. This multilayer microstructure integrates independent photonic and magnetic functionalities into a mobile, embeddable platform that can be fabricated at scale and offers a versatile, highly tunable photonic design space.

## 2 Results

### 2.1 Design of Magneto‐Photonic Metaparticles

We realize this design experimentally by fabricating magneto‐photonic metaparticles that embody the proposed integration of magnetic actuation and photonic functionality. As shown in Figure 1b, each magneto‐photonic metaparticle consists of a polymer base, a magnetic core, a nanoimprinted polymer scaffold, and a top layer. This multi‐layered architecture allows the magnetic core to govern the locomotion and orientation, while the nanoimprinted surface and the top layer create and enhance photonic functionality, respectively. The magnetic core is shaped as an isosceles triangle to break radial symmetry and introduce shape anisotropy as shown in Figure S1, which promotes alignment during rotation and allows the rotational motion to be easily visualized. The fabrication process, shown in Figure 1d, assembles magnetic and photonic layers. Here, SU‐8, a negative photoresist, serves as a polymer scaffold, enabling the particle body to be formed by photolithography and the photonic surface to be patterned by nanoimprint lithography [42]. Arrays of SU‐8 bases are first fabricated on a silicon wafer. The magnetic core is then integrated through three sequential steps: (1) spin‐coating and developing a positive photoresist on top of the SU‐8 base, (2) directional deposition of a ferromagnetic material using an e‐beam evaporator, and (3) lift‐off of the photoresist. Because the patterning occurs within the recessed region of the polymer base, the positive photoresist is made sufficiently thick to cover the base and is developed in an inverted pattern, defining the magnetic core geometry. Subsequent metal deposition and lift‐off leave the magnetic core precisely positioned on top of the SU‐8 base. The top SU‐8 layer is then formed by spin‐coating and developing, followed by nanoimprinting photonic patterns and a deposition of a dielectric or metallic coating. Finally, magneto‐photonic microstructures are detached from the substrate and collected.

### 2.2 Controlled Locomotion and Rotational Motion of Magnetic Metaparticles

To demonstrate the magnetic actuation and locomotion capabilities of the magneto‐photonic microdiscs, we first consider the expected scaling of translational motion under an external magnetic field. At distances larger than the characteristic size of the magnet, the magnetic field *B* can be approximated as that of a point dipole (*B* ∝ *d*_m‐p_^−3^, where *d*_m‐p_ denotes the magnet‐particle distance), resulting in a magnetic force that scales as *F*_mag_ ∼ ∇(*m*·*B*) ∝ *d*_m‐p_^−4^, where *m* is the magnetic moment of the particle. Under low‐Reynolds‐number conditions, the translational velocity is governed by viscous drag (*F*_drag_ ∼ *γv*), where *γ* is an effective hydrodynamic drag coefficient that depends on fluid viscosity, particle geometry, and near‐wall confinement. Balancing magnetic force and viscous drag therefore yields the expected scaling of *v* ∝ *d*_m‐p_^−4^ (Section 4.3).

**FIGURE 2:**
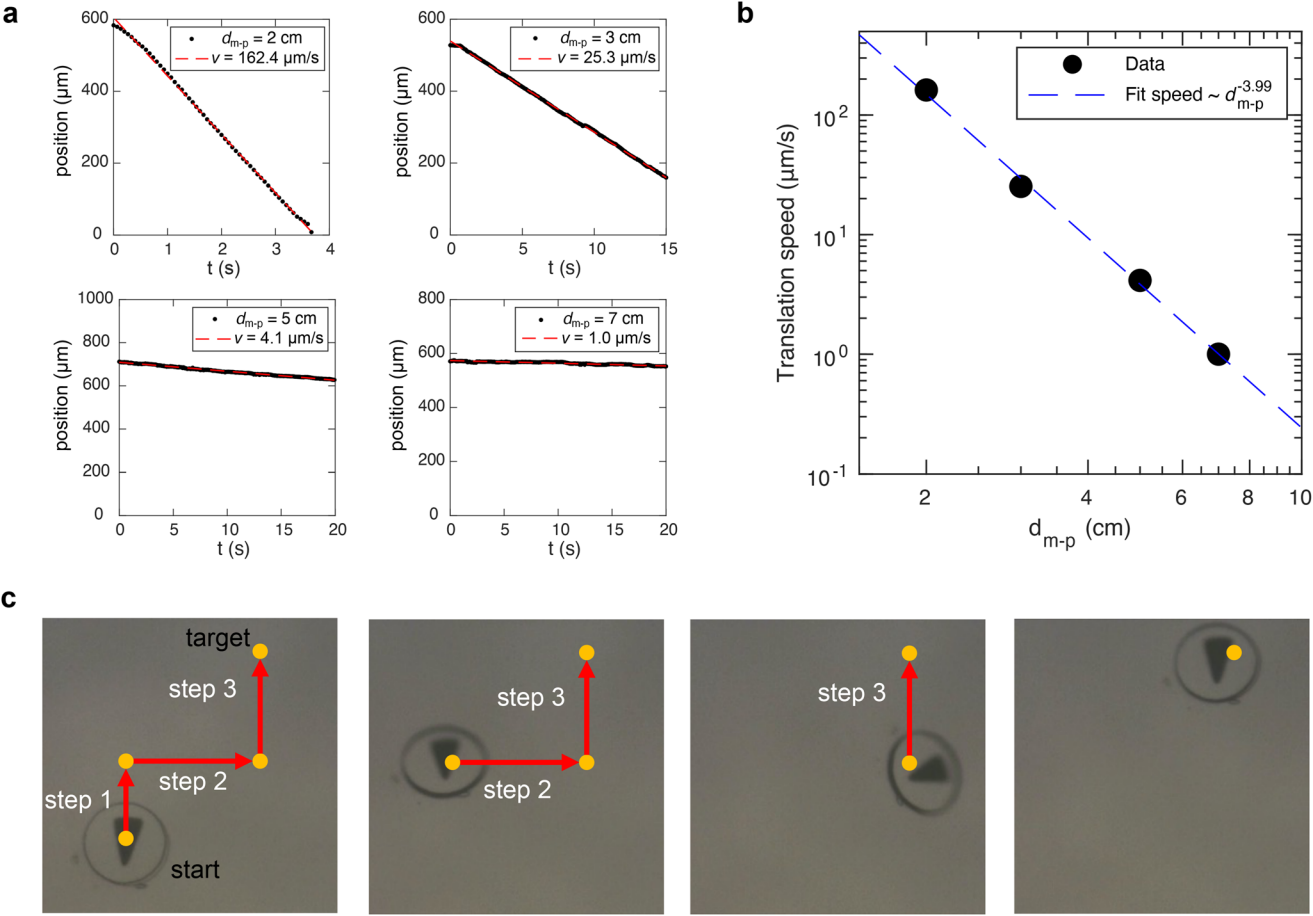
Controlled locomotion of magnetic metaparticles. (a) Measured velocities of magnetic microdiscs as a function of the magnet‐particle distance (*d*_m‐p_) from the magnet. (b) Log‐log plot of measured velocity (𝑣) as a function of the magnet‐particle distance (*d*_m‐p_). The trend is consistent with the 𝑣 ∝ *d*_m‐p_^−4^ scaling, predicted from the far‐field magnetic dipole gradient ($dB/d\left(d_{m-p}\right)\propto {d_{m-p}}^{-4}$) and viscous drag balance for a saturated ferromagnetic inclusion in low‐Reynolds‐number flow. (c) Sequential locomotion of a magnetic particle programmed to follow a series of translational motions (see also Movie S1).

To experimentally validate this behavior, a neodymium magnet was used to actuate the microdiscs immersed in DI water. The position of each microdisc was tracked over time to determine the translation speed (*v*) as a function of the magnet‐particle distance (*d*_m‐p_). Figure 2a shows representative single‐particle measurements, with velocities of 162.4, 25.3, 4.1, 1.0, and 0 µm/s for *d*_m‐p_ = 2, 3, 5, 7, and 10 cm, respectively. At *d*_m‐p_ = 10 cm, the microdisc exhibited negligible displacement over 20s, making its velocity difficult to measure. As shown in Figure 2b, the log‐log plot of velocity versus distance revealed a slope of −3.99, in good agreement with the predicted 𝑣 ∝ *d*_m‐p_^−4^ scaling. While the regimes are plotted as a function of *d*_m‐p_, they can be interpreted in terms of magnetic field, which governs the magnetic driving force. For the neodymium magnet used in this study, the on‐axis magnetic field decreases from approximately 53 mT to 1.0 mT for *d*_m‐p_ = 2−10 cm. The observed motion regimes therefore correspond to approximate field thresholds that can be used to interpret actuation behavior across different setups. To demonstrate programmable control, the microdisc structures were actuated through a sequence of three translational motions: (1) upward, (2) rightward, and (3) upward again, by sequentially switching the magnet's position, as shown in Figure 2c and Movie S1. These results confirm that the magneto‐photonic microdiscs exhibit controllable, programmable locomotion, and enable obstacle avoidance and targeted movement under simple magnetic field manipulation.

**FIGURE 3:**
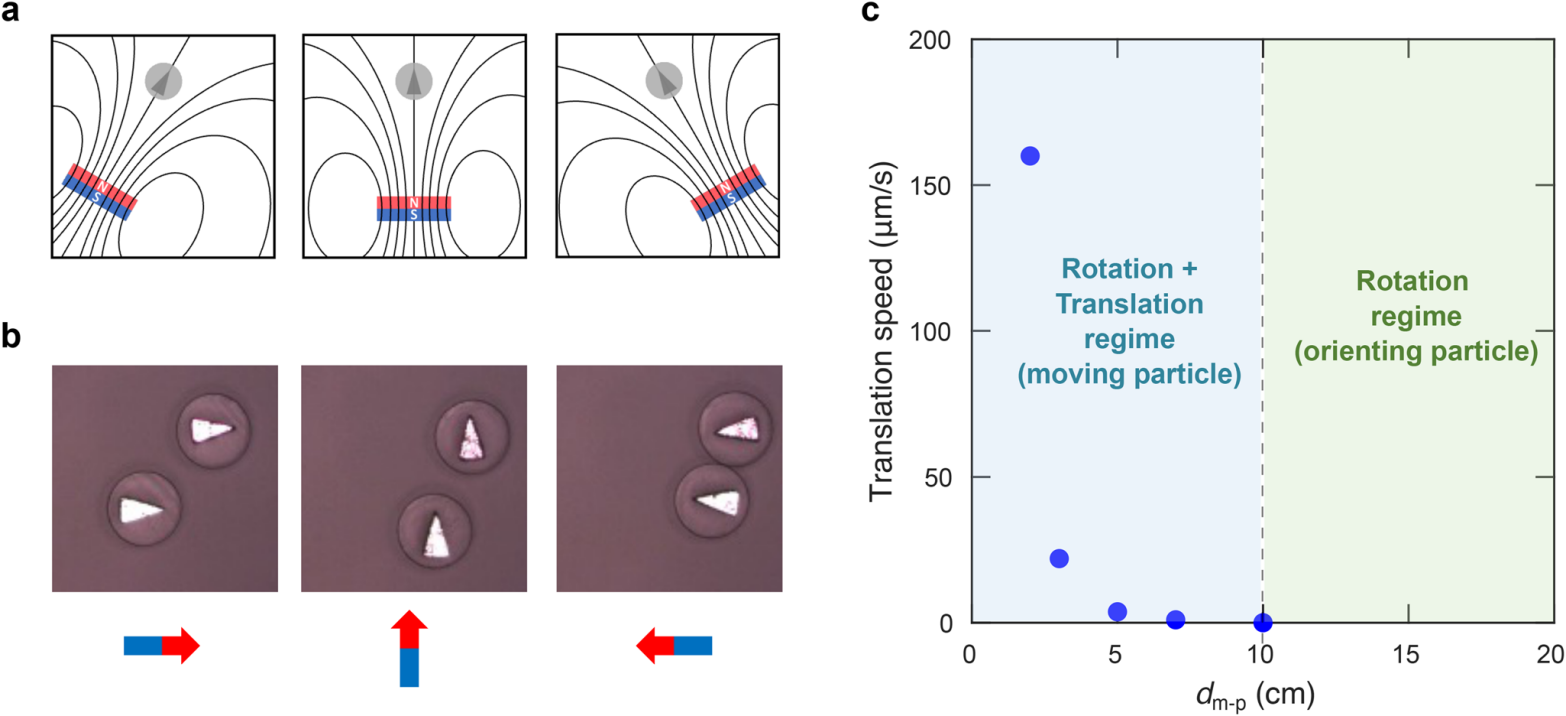
Rotational motion and field‐dependent motion regimes of magnetic metaparticles. (a) Illustration of particle alignment under different magnet orientations and positions. (b) Actual alignment of particles under different directions of the applied magnetic field (arrows indicate field direction). (c) Distinct motion regimes are controlled by the field strength: moving (high‐field/closer proximity to the magnet) versus orienting (low‐field/larger distance to the magnet) (see also Movie S2).

Next, we show that the magneto‐photonic microdiscs can be both translated and rotated on demand. We implement this functionality through two sequential magnetic motions: (1) translating the metaparticle to the desired location, as shown in Figure 2c, and (2) rotating it to achieve the target beam steering direction, as shown in Figure 3a,b, and Movie S2. The metaparticle can be oriented reproducibly and precisely at discrete angular positions in 5° increments, as shown in Figure S2. To estimate the dynamic response of the rotational actuation, we tracked the angular orientation of both the external magnet and the metaparticle from synchronized videos. The metaparticle follows the imposed magnetic rotation with a finite delay, corresponding to a measurable phase lag that increases with angular speed. At low angular speeds, the variation of phase lag remains small. As shown in Figure S3, at magnet rotation rates above 135 rpm, the particle rotation begins to show an increased variation of phase delay. This desynchronization response may not be solely limited by viscous drag, and additional factors such as substrate interaction could influence the rotation dynamics.

For our particle design, the magnet‐particle distance (*d*_m‐p_) can separate motion regimes. In regime I (*d*_m‐p_ < 10 cm), the applied field is sufficiently strong to support locomotion (i.e., the first magnetic motion), translating the particle in the desired direction. In regime II (10 cm < *d*_m‐p_ < 20 cm), the applied field can only support rotation: this regime is suitable for beam steering, or second magnetic motion, where the rotation is decoupled from translation (Figure 3c). The magnetic metaparticle is oriented toward/away based on the applied field direction due to the built‐in magnetic shape anisotropy, as shown in Figures 2c and S1. Finally, in regime III (*d*_m‐p_ > 20 cm), the field is too weak to effectively orient the particle (i.e., the rotational rate decreases below 6 rpm). Together, these field‐dependent regimes establish a straightforward mechanism to selectively trigger translation or rotation, providing precise and decoupled control necessary for active beam steering.

### 2.3 Experimental Demonstration of Active Beam Steering Capability

**FIGURE 4:**
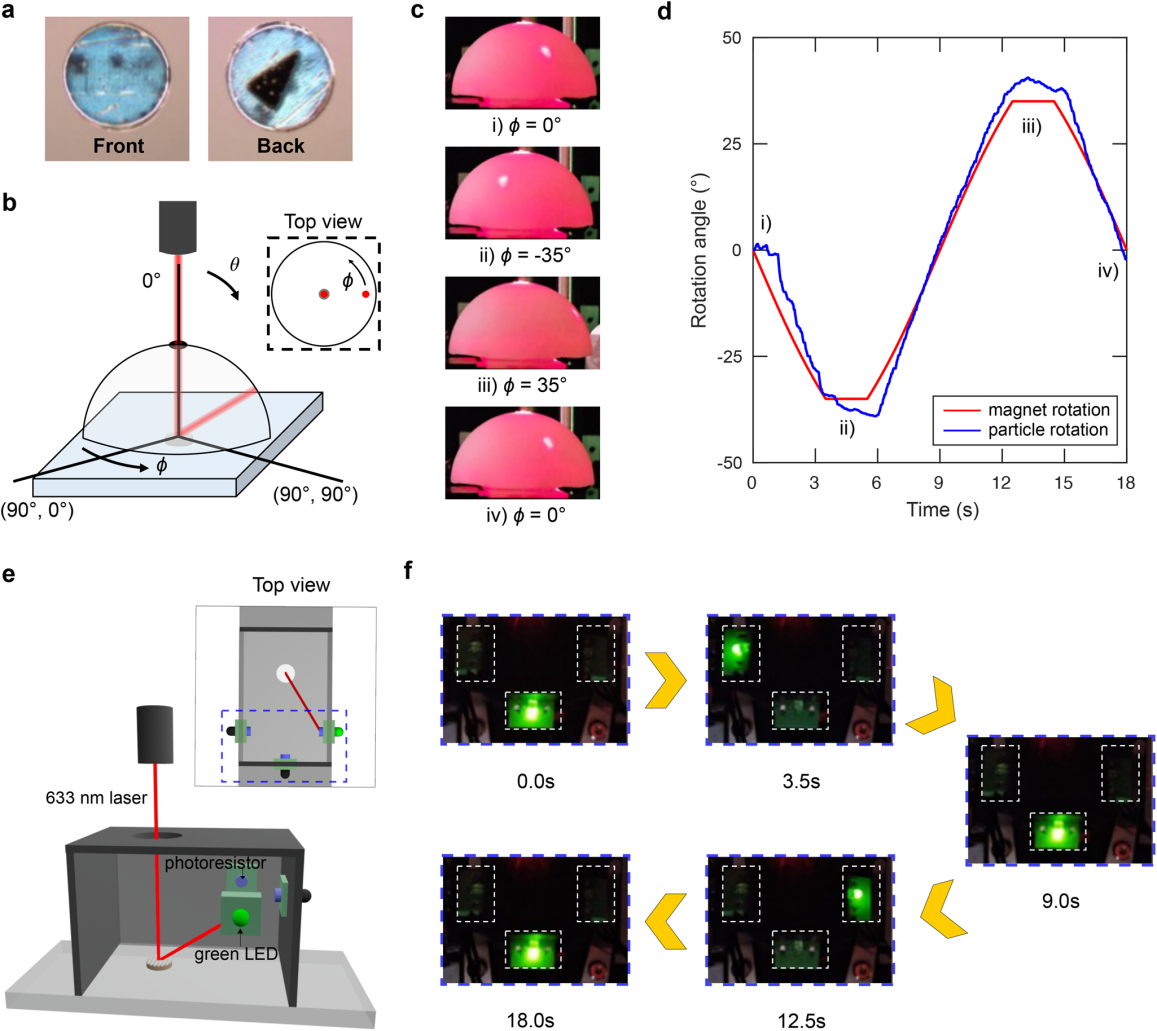
Integration of optical functionality and demonstration of active beam steering. (a) OM images of magneto‐photonic microdiscs with a nanoimprinted grating surface. (b) Experimental setup for characterizing active light‐steering behavior under controlled circular magnet motion. (c) Trajectories of the steered beam as the microdisc is rotated by 35° clockwise, 70° counterclockwise, and again 35° clockwise. (d) Synchronized response of particle rotation and magnet rotation (Movie S4). (e) Setup for real time beam‐steering demonstration: a container with edge light sensors (inward‐facing photodetectors and outward‐facing indicator LEDs). When the steered beam from the magneto‐photonic particle strikes a light sensor, the corresponding LED is activated. (f) Top view of the setup in (e), showing the progression of beam steering under programmed magnet rotation of a particle immersed in water (see also Movie S5).

To integrate photonic functionality into the metaparticle, we employ nanoimprinting, a scalable fabrication approach that leverages the deformable nature of SU‐8 to enable high‐throughput replication of nanoscale features. To improve yield and minimize detachment of the SU‐8 film, we imprint the mold onto the metaparticle surface where the SU‐8 film has been cross‐linked. As a fabrication proof‐of‐concept, we create photonic particles by imprinting a ruled grating (pitch 833 nm, see Section 4.2) and depositing 50 nm Ag, as shown in Figures 4a and S5. Diffraction efficiency measurements show strong agreement with simulations, indicating both the high quality of the nanoimprint process and high fidelity of the RCWA simulation, as depicted in Figure S6e,f. Based on this, for metaparticles placed in water, we estimate a high diffraction efficiency of 0.7 for the ‐1^st^ order for both TM and TE polarization, as shown in Figure S6a,b. Figure S6c further shows a high degree of polarization insensitivity for any mixture of TE/TM polarization. Furthermore, this design is robust to misalignment in incident angle, as shown in Figure S6d.

Once the photonic pattern is imprinted, the magnetic and photonic orientation are synchronized, such that in‐plane rotation of the metaparticle under external magnetic field determines the direction of beam steering. The steering angle exhibits a mean error of 0.57° from the target value, with a standard deviation of 0.12°, indicating high repeatability with a small systematic offset. We note that, even if the metaparticle is accidentally oriented with its photonic surface facing downward, an out‐of‐plane magnetic rotation can flip it, restoring the active surface to the upward orientation, as shown in Figure S4 and Movie S3.

Having fabricated the composite magneto‐photonic particle, we demonstrate its beam‐steering capability by tracking the trajectories of light during controlled magnetic rotation. Figure 4b shows immersed metaparticles placed beneath a hollow, translucent hemisphere with an aperture at the top. A neodymium magnet, mounted on a linearized stepper motor via an optical dovetail rail, was positioned so that its linear motion was converted into circular motion around the sample. A red laser beam (633 nm) was directed through the aperture onto the sample plane, and the directed beam spot was projected onto the inner surface of the hemisphere. For in‐plane rotation, the magnet was oriented toward the metaparticle while moving circularly, producing a visible trajectory of the steered beam along the azimuthal direction, as shown in Figure 4b. The magnet was programmed to execute a sequence of motions: (1) 35° clockwise rotation, (2) 2 s pause, (3) 70° counterclockwise rotation, (4) 2 s pause, and (5) 35° clockwise rotation. The diffracted beam spot followed these programmed rotations immediately and without delay, confirming that the photonic orientation precisely tracks the magnetic orientation of the particle, as shown in Figure 4c,d and Movie S4.

Observing that the magnetic and photonic orientations are synchronized, we proceed to demonstrate active beam steering of the magneto‐photonic metaparticle. The experimental setup consists of three light‐sensor boards, mounted on the three walls of the container, with the light sensors facing inward. On each board, opposite of the light sensor, an outward facing LED indicates whether the corresponding sensor is triggered (as shown in Figure 4e). Red laser light, directed through a small aperture in the container top, is steered by the metaparticle with its azimuthal steering angle controlled by the circular motion of an external magnet. The light‐sensor boards are programmed to activate the corresponding green LED whenever the associated sensor is triggered, serving as an indicator that the steered beam has hit the target sensor. Using the same programmed sequence of circular magnet motions as in Figure 3d, this setup produces sequential LED activations, as demonstrated in Figure 4f and Movie S5. These results validate real time, magnetically driven beam steering and demonstrate the rapid and synchronized response of the magneto‐photonic metaparticle to external magnetic fields.

### 2.4 Nanoimprinting‐Compatible Optimized Metagratings Design

**FIGURE 5:**
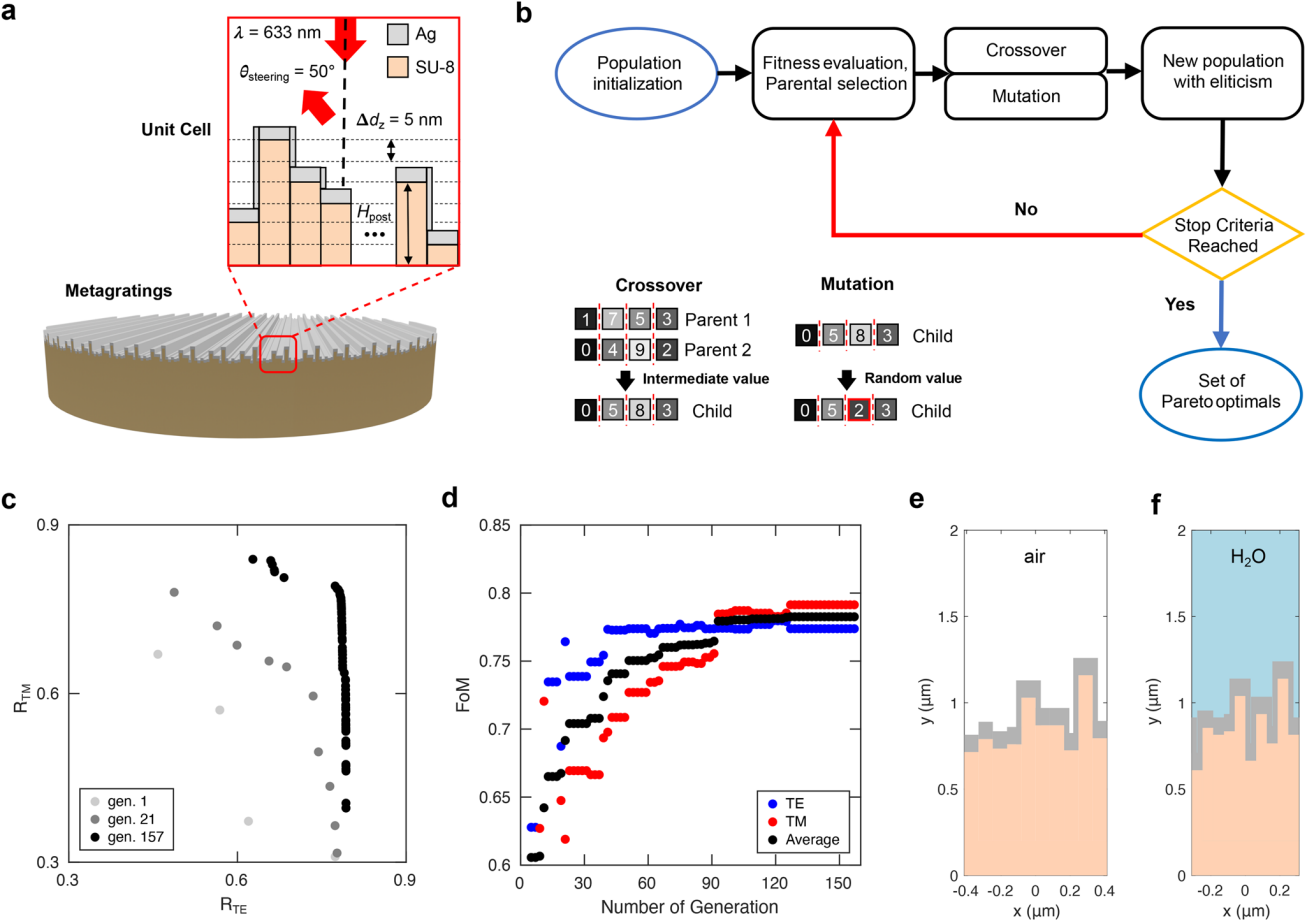
Nanoimprint‐compatible design of metaparticles for polarization‐insensitive light steering. (a) Illustration of a metagrating‐engraved metaparticle and unit cell structure consisting of polymeric (SU‐8) posts with a metal (Ag) overlayer. Wavelength is 633 nm and target steering angle is 50°. (b) Flow chart of a genetic‐algorithm‐implemented multi‐objective optimization. (c) Evolution of the Pareto front at the first, 21^st^, and 157^th^ generations of the genetic multi‐objective optimization. (d) Multi‐objective optimization over successive generations, with the Figure of merit defined by the knee point that minimizes the distance to the ideal point (TE, TM) = (1, 1). The average steering efficiency shows a gradual increase over generations. (e) Optimized unit cell in air (*n* = 1.0) achieving steering efficiencies of 0.77 (TE) and 0.79 (TM). (f) Optimized unit cell in water (*n* = 1.33), achieving efficiencies of 0.76 (TE) and 0.79 (TM). These results are obtained from RCWA simulations; experimental realizations are discussed in Section 4.

Building on the proof‐of‐concept demonstrations with a simplified imprinted grating, we next extend our approach toward a nanophotonic design that fully exploits the fabrication scalability offered by nanoimprint lithography while remaining compatible with fabrication constraints. The results in this section are derived from numerical simulations, and possible experimental realizations are discussed in Section 3 below. Achieving high‐performance metaparticle nanophotonic designs compatible with nanoimprint processes is a key challenge. Although nanoimprint lithography enables large‐area fabrication of photonic structures, its reliance on polymer materials with low to moderate refractive indices limits achievable optical efficiency. We address this challenge by designing a multi‐level photonic unit cell with a metallic overlayer and optimizing it for polarization‐independent beam steering. Figure 5a depicts a unit cell design consisting of an array of polymeric posts with identical widths but varying heights, over which a metallic layer is deposited. Considering the directional nature of material deposition, the geometry was tailored such that the metallic overlayer slightly extends over the sidewalls of the posts as well as their top surfaces. Here, the metallic overlayer plays a role in enabling efficient reflective operation and delivers high steering efficiency by nonresonant phase engineering, as demonstrated in Figure S9. Next, we formulate a multi‐objective photonic design optimization aimed at maximizing steering efficiencies under both TE and TM polarizations. This dual‐polarization optimization is key, as the orientation of the photonic surface dynamically changes due to the rotation of the magneto‐photonic metaparticle, resulting in a superposition of TE and TM components in the incident light. We employ a genetic algorithm (GA) to perform the optimization and use rigorous coupled‐wave analysis (RCWA) to calculate steering efficiency. As illustrated in Figure 5b, the GA evolves a population of candidate structures by evaluating their fitness with respect to both objectives, selecting high‐performing designs, and generating new candidates through crossover and mutation. This process continues until convergence toward a Pareto‐optimal set of solutions where improvements in one objective cannot be achieved without compromising the other. Among the final candidates, the design with the maximum product of efficiencies under TE and TM polarizations was selected.

For our design, the parameters were set for an operating wavelength of 633 nm, a steering angle of 50°, and ten posts per unit cell whose heights served as the optimization variables (Figure 5a). This optimization method generates a set of solutions at each generation and progressively evolves the Pareto front, driving the steering efficiency toward unity for both TE and TM modes. As shown in Figure 5c, from the Pareto front at each generation, the knee point is selected as the optimal solution by minimizing the distance to the ideal point (TE, TM) = (1, 1). This optimal solution steadily increased and eventually converged over successive generations. Because the optimization simultaneously targets high steering efficiency for both TE and TM polarizations, the evolution of their Pareto front reflects the trade‐off surface between the two objectives. The evolution of the Pareto front and the growing number of optimal (rank‐1) candidates further confirm the effective progression of the multi‐objective optimization, as shown in Figure S7. Figure 5e shows that the resulting optimized structure in air achieves steering efficiencies of 0.77 and 0.79 for TE and TM modes, respectively. These efficiencies are significantly higher than for conventional gratings: for comparison, a conventional ruled grating with a blazing angle of 25° yields a lower efficiency of 0.75 for TM mode, and a much lower efficiency of ∼0.35 for TE mode. This highlights the superior polarization‐independent performance of the optimized structure enabled by our design and fabrication strategy. Furthermore, Figures 5f and S8 show that the optimized unit cell design retains high steering efficiencies of 0.76 (TE) and 0.79 (TM) even in an aqueous environment (*n* = 1.33). This highlights the versatility of our magneto‐photonic design for high‐performance beam steering in different environments.

## 3 Discussion

We have introduced a magneto‐photonic metaparticle as a mobile, field‐programmable platform for real time beam steering, bridging the gap between static, substrate‐bound metasurfaces and mobile particle scatterers with limited optical functionality. A key feature of this architecture is the decoupling of mechanical and photonic functionalities: the magnetic core governs rigid‐body locomotion and orientation, while the nanoimprinted surface provides optical functionality that is designed independently of the actuation mechanism. This decoupling makes the optical design space independent of the mechanical platform, accommodating arbitrary nanoimprint‐compatible designs, including metagratings for high‐efficiency, polarization‐insensitive light steering. Experimental results demonstrate programmable translation and in‐ and out‐of‐plane rotation, enabling dynamic beam steering in an aqueous medium in direct response to an applied magnetic field. The synchronized magnetic and photonic orientations enable the metaparticle to function as a freely positionable, magnetically reconfigurable micro‐optical element capable of real time, directional light control.

We envision several promising directions stemming from this work. The large degree of freedom in designing photonic surfaces can enable a wide range of photonic functionalities. While this study focuses on reconfigurable beam steering, the same platform could be extended to beam focusing or diverging for microlens applications, as well as to spectral, polarization, and emission control for micro‐filter applications. More broadly, this platform connects to the wider context of structured‐wave steering and routing concepts [43], with the distinct feature that the optical element is mobile and its orientation is magnetically reconfigurable rather than fixed. Imprinting provides both the scalability of nanoscale patterning and the capacity to replicate 3D surface structures [42]. The accessible design space extends well beyond the experimental proof‐of‐concept gratings used here, encompassing sharp or curved surfaces and multi‐level metastructures such as the proposed designs in Section 2.4. The required nano‐imprint master can be fabricated via grayscale lithography [44] or multi‐step e‐beam writing [45].

We note that the presented platform should be viewed as a proof‐of‐concept demonstration and not an application‐ready device. However, the metaparticle design is inherently scalable, and with the appropriate control of the photonic pattern size, the structure could be miniaturized to tens or even several micrometers. With further materials and device development, such structures could become relevant for biophotonic applications, such as bioimaging and sensing, targeted photothermal heating, and photodynamic therapy [46, 47]. With regards to materials selection, crosslinked SU‐8 or parylene C can serve as a biocompatible barrier [48, 49], the metallic layer could be passivated or replaced by a more chemically stable metal (e.g., gold) or dielectric, and the magnetic core could be replaced by more biocompatible ferromagnetic materials (e.g., Fe_3_O_4_ [50]). In addition to such applications, mobile magneto‐photonic particles may also be relevant for reconfigurable optical routing, visible light communication, and magnetically reconfigurable photonic elements in lab‐on‐chip or on‐chip optical systems [51, 52].

## 4 Experimental Section

### 4.1 Fabrication of Magnetic Microdiscs

A diluted solution of negative photoresist (SU‐8 2010, MicroChem) was spincoated onto a silicon wafer to form an 800‐nm‐thick SU‐8 bottom layer. A photomask consisting of square arrays of 100‐µm‐diameter circles was used to regioselectively irradiate the SU‐8 film with the mask aligner (MABA6, SUSS). SU‐8 developer was used to create SU‐8 microdiscs as the bottom layer. An isosceles triangular cobalt core with base, height, and thickness of 40 µm, 70 µm, and 50 nm are then deposited onto SU‐8 microdiscs using the lift‐off process. SU‐8 is spincoated and developed onto a cobalt‐deposited SU‐8 microdiscs to create magnetic microdiscs (10 µm thickness). The samples were then immersed inside the MF‐319 solution to detach magnetic microdiscs from a substrate.

### 4.2 Fabrication of Magneto‐Photonic Metaparticles

Before detaching magnetic metaparticles, surfaces of SU‐8 microdiscs are imprinted with reflective ruled gratings (pitch 3.33 µm or 833 nm, Thorlabs) at glass transition temperature of SU‐8 and 300 psi for 300s (NX B200, Nanonex). A 50 nm thick layer of Ag was deposited using an e‐beam evaporator (CHA Industries). The samples were then immersed inside the MF‐319 solution to detach metaparticles from a substrate.

### 4.3 Magnetic Actuation Procedure of Magnetic Metaparticle Rotation

To facilitate magnetic control of rotation of a magnetic microdisc, a neodymium magnet (max energy product: 263–287 kJ/m^3^) is precisely manipulated to induce in‐plane and out‐of‐plane rotation motion of the microdisc. In‐plane rotation is actuated by moving the magnet in a circular trajectory around the center of the microdisc while gradually rotating the magnet to make magnetic field continuously face the microdisc. However, when the microdisc is in contact with the substrate, frictional forces can be high enough to prevent in‐plane rotation, even when appropriate magnetic actuation is applied. In such cases, out‐of‐plane rotation can serve to momentarily lift the disc off the substrate, reducing contact and enabling smoother in‐plane rotation. Out‐of‐plane rotation is actuated by fixing the position of the magnet and rotating it around its central axis, which is parallel to the substrate surface.

### 4.4 Characterization

Images of microdiscs and rotational motions of magnetic microdisc were acquired using optical microscopy in reflection mode, where a 5× and 10× objective lens with a numerical aperture of 0.30 and 0.45, respectively, were used. Magnetic moments of magnetic cores at different orientations are characterized with vibrating‐sample magnetometer.

### 4.5 Simulation and Optimization

Calculations on diffraction efficiencies and diffraction orders were performed with the RCWA method, using an open‐source software package [53]. Multi‐objective optimization was performed with Global Optimization Toolbox implemented in MATLAB.

## Conflicts of Interest

The authors declare no conflicts of interest.

## Supporting information




**Supporting File 1**: adma74028‐sup‐0001‐SuppMat.pdf.

**Supporting File 2**: adma74028‐sup‐0002‐MovieS1.mp4.

**Supporting File 3**: adma74028‐sup‐0003‐MovieS2.mp4.

**Supporting File 4**: adma74028‐sup‐0003‐MovieS3.mp4.

**Supporting File 5**: adma74028‐sup‐0004‐MovieS4.mp4.

**Supporting File 6**: adma74028‐sup‐0005‐MovieS5.mp4.

## Data Availability

The data that supports the findings of this study are available in the Supporting Information of this article.
