# Supplementary material for: Field‐Programmable Mobile Magneto‐Photonic Metaparticles for Active Light Manipulation and Steering
Source: Adv Mater. 2026 Jul 15;38(47):e74028. doi: 10.1002/adma.74028 (PMC13496083; doi:10.1002/adma.74028)
Supplement: Supplementary file 1 [file ADMA-38-e74028-s001.pdf]

Supplementary Information for

Field-Programmable Mobile Magneto-Photonic Metaparticles for Active Light Manipulation and Steering

Seung Yeol Lee[1], Yujie Luo[1], Ognjen Ilic[1]

[1] Department of Mechanical Engineering, University of Minnesota, Minneapolis, 55455 USA

Table of Contents

S1. Description for Supporting Movies (Movie S1 – S5)

S2. Magnetic Properties of a Cobalt Core

S3. In-Plane Rotation of Magnetic Metaparticles

S4. Out-of-Plane Rotation of Magnetic Metaparticles

S5. Optical Properties of Magneto-Photonic Metaparticles

S6. Multi-Objective Optimization of Metagratings Using Genetic Algorithm

S7. Mechanism of the Metallic Overlayer and Sidewalls

S8. Comparison of Optical Microsystems

S1. Description for Supporting Movies (Movie S1 – S5)

Movie S1: Sequential translation of magnetic metaparticle
Movie S2: In-plane and out-of-plane rotation of magnetic metaparticle
Movie S3: In-plane and out-of-plane rotation of magneto-photonic metaparticle
Movie S4: Synchronized photonic and magnetic orientations of a magneto-photonic metaparticle
Movie S5: Active beam steering demonstration

S2. Magnetic Properties of a Cobalt Core

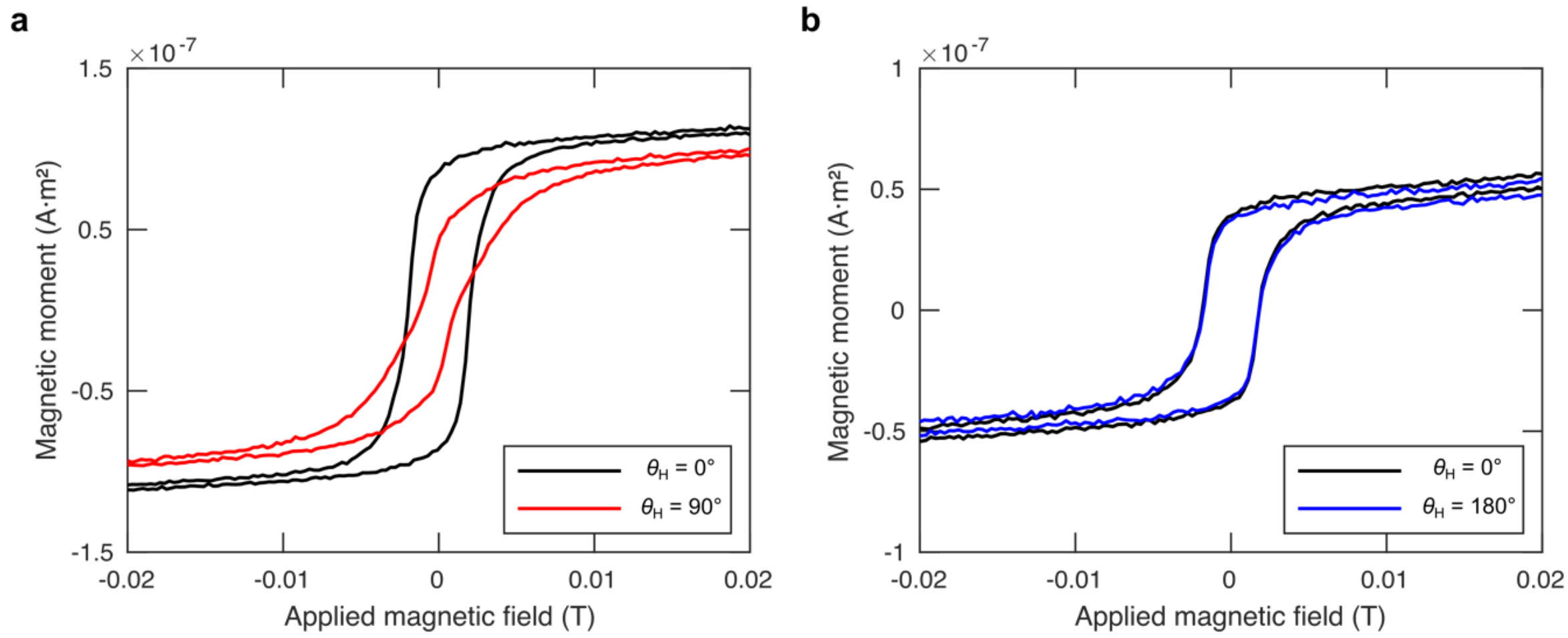

Figure S1. Magnetic properties of arrays of cobalt cores depending on the angle between applied magnetic field and symmetry axis of the cobalt core, θ_H. All cobalt cores are aligned on top of a Si wafer. (a) Plot of magnetic moment versus applied magnetic field for $\theta_H = 0°$ And $\theta_H = 90°$, showing different hysteresis loops, which comes from magnetic anisotropy. (b) Plot of magnetic moment versus applied magnetic field for $\theta_H = 0°$ And $\theta_H = 180°$, showing similar hysteresis loops, which indicates that two orientations can coexist under applied magnetic field.

S3. In-Plane Rotation of Magnetic Metaparticles

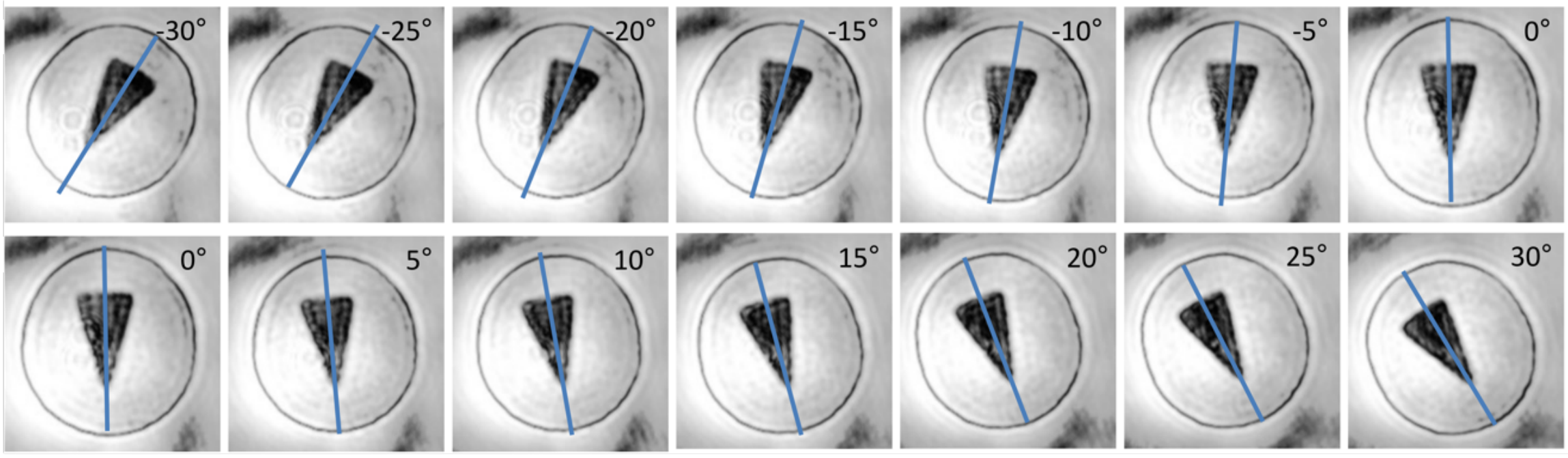

Figure S2. In-plane rotation of magnetic metaparticle from -30 degree to 30 degree with every 5-degree increment.

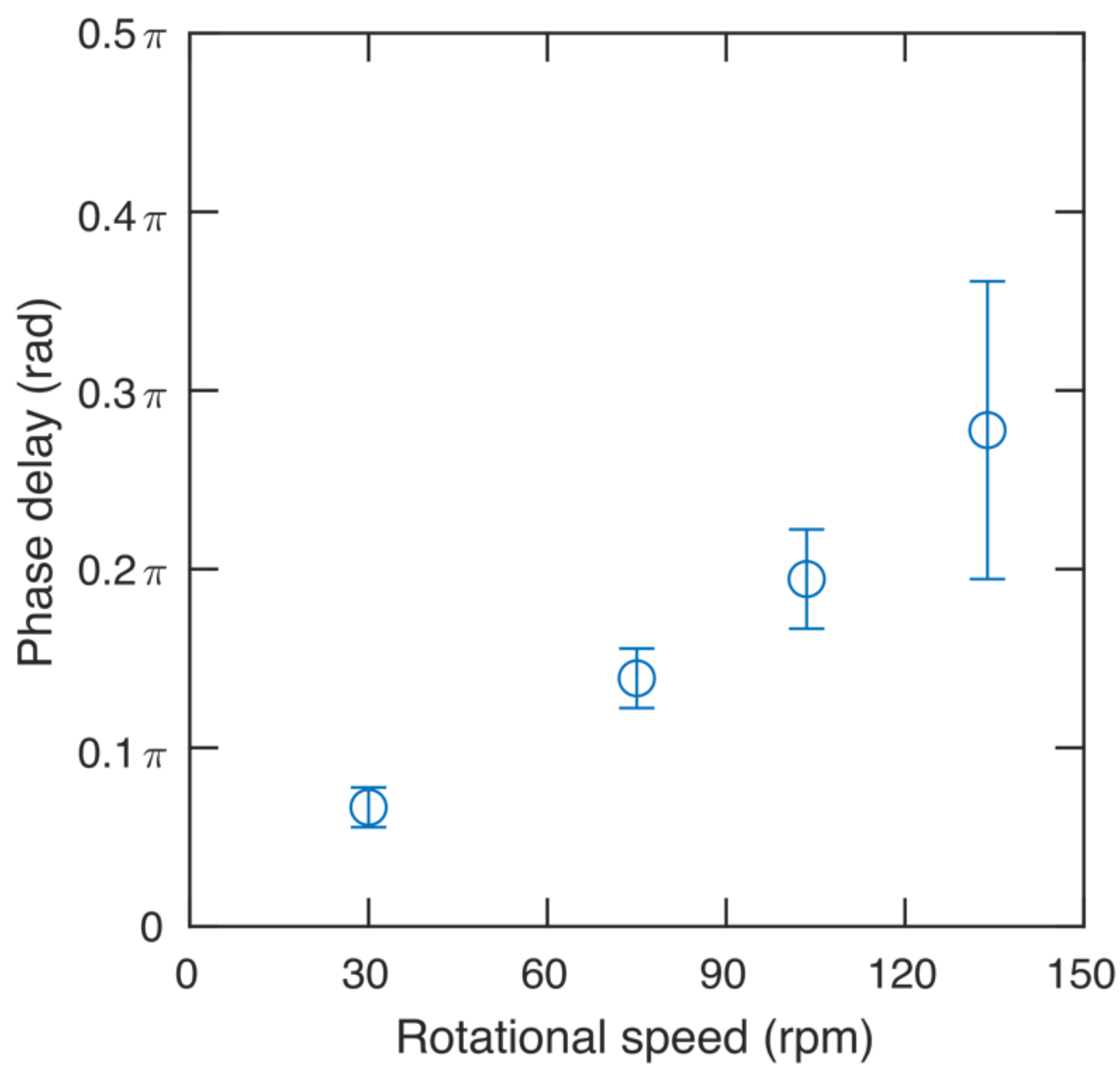

Figure S3. Phase delay depending on the angular velocity of the metaparticle where the phase delay is defined as the product of the rotational speed and the response time, assuming constant rotational speed. Response time of the magnetic metaparticle is around 0.06s, and the phase delay increases almost linearly as rotational speed increases. Rotational speed over 135 rpm shows large variance in phase delay, implying unstable phase lag due to stick-slip behavior.

S4. Out-of-Plane Rotation of Magnetic Metaparticles

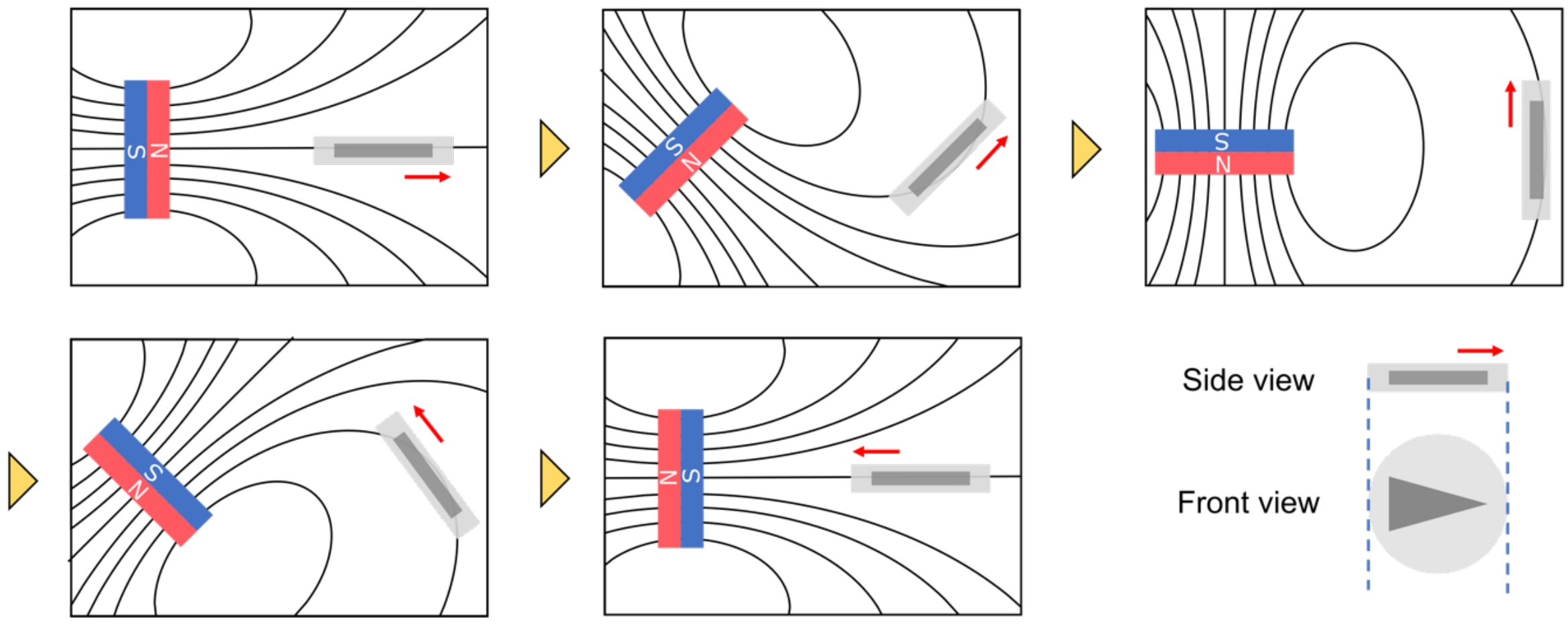

Figure S4. Illustrations of methods to manipulate in-plane rotation of a magnetic particle. Similar magnet motion with fig. S2b, but with out-of-plane magnet rotation, the particle will rotate out-of-plane in opposite direction and the particle will eventually flip upside down.

S5. Optical Properties of Magneto-Photonic Metaparticles

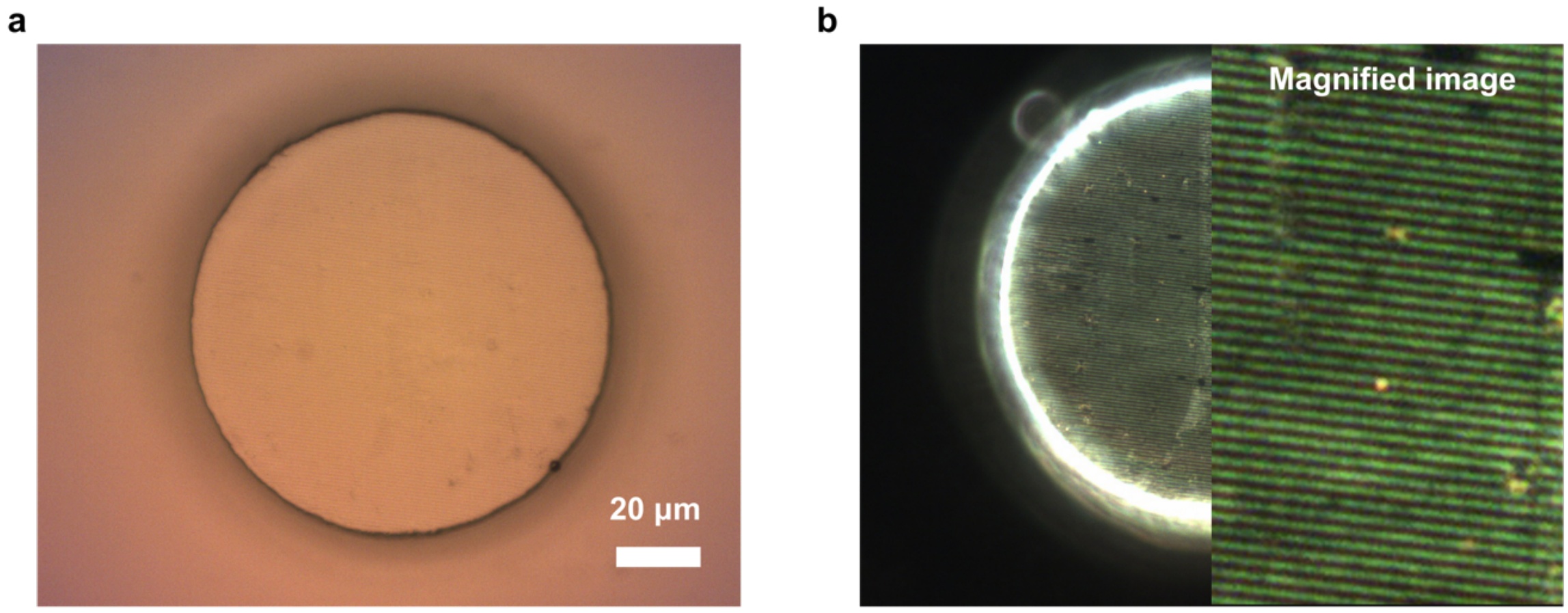

Figure S5. A magneto-photonic metaparticle for active beam steering demonstration. OM images of a magneto-photonic metaparticle (a) at bright field and (b) at dark field. Magnetic core is incorporated inside the microdisc and the microdisc is imprinted with ruled gratings with 1200 grooves/mm, which diffracts light only at 1st and -1st orders with 49.4° steering angle. Magnified image from the dark field shows grooves imprinted on the metaparticle.

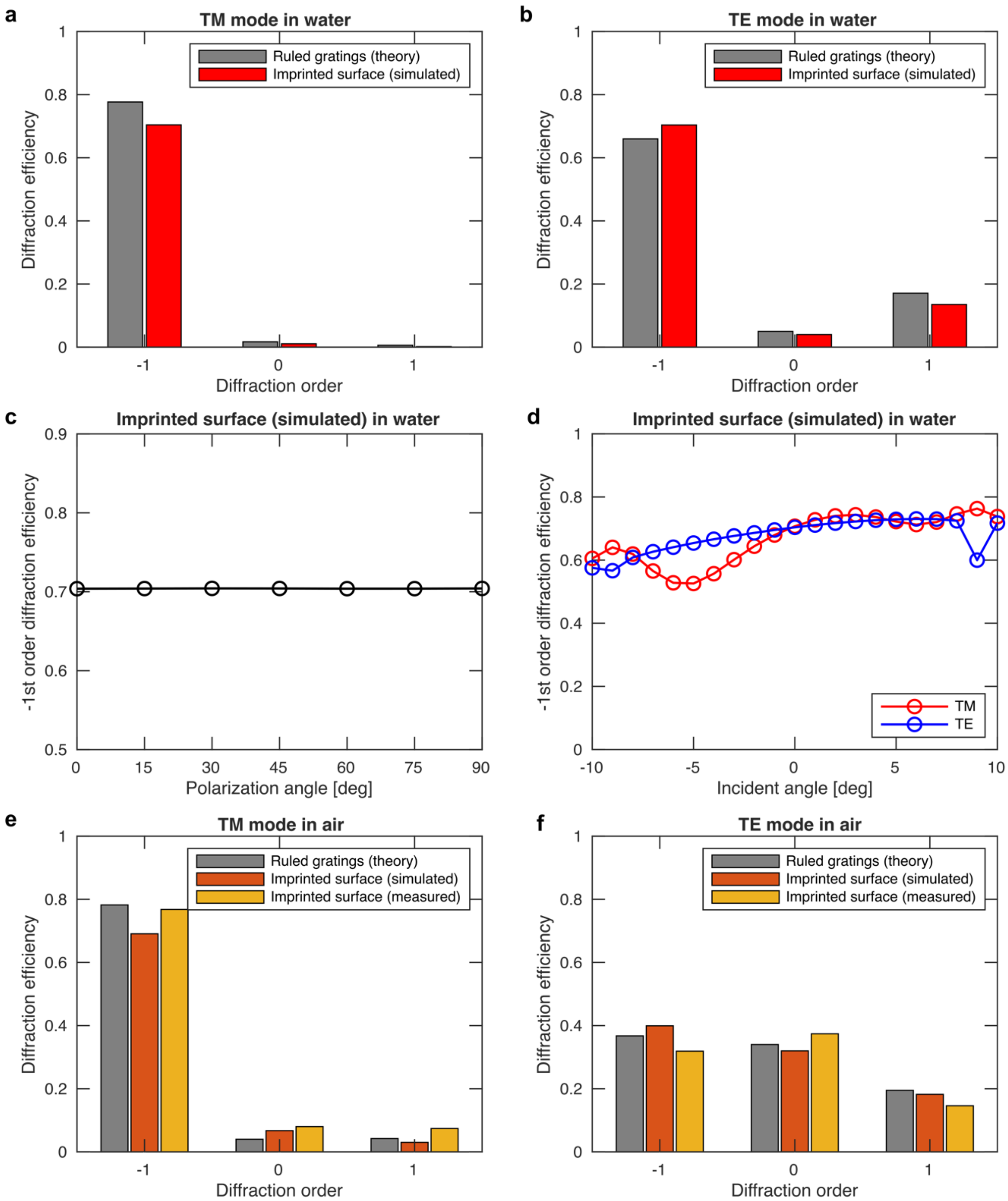

Figure S6. Diffraction efficiencies of imprinted photonic metaparticles, -1st order being desired signal, and 0th and 1st orders being unwanted signals. Diffraction efficiencies of ruled gratings (theory) and imprinted surface (simulated) in water at (a) TM mode and (b) TE mode, showing desired efficiencies over 0.7 for both TM and TE modes. Diffraction efficiencies of imprinted surface (simulated) in water (c) over polarization angle for polarization sensitivity check and (d) over incident angle for incident angle misalignment check. This shows insensitivity of the imprinted surface for polarization angle and incident angle. Diffraction efficiencies of ruled gratings (theory), imprinted surface (simulated), and imprinted surface (measured) in air at (e) TM mode and (f) TE mode, which verifies high fidelity of matching between measured and simulated diffraction.

S6. Multi-Objective Optimization of Metagratings Using Genetic Algorithm

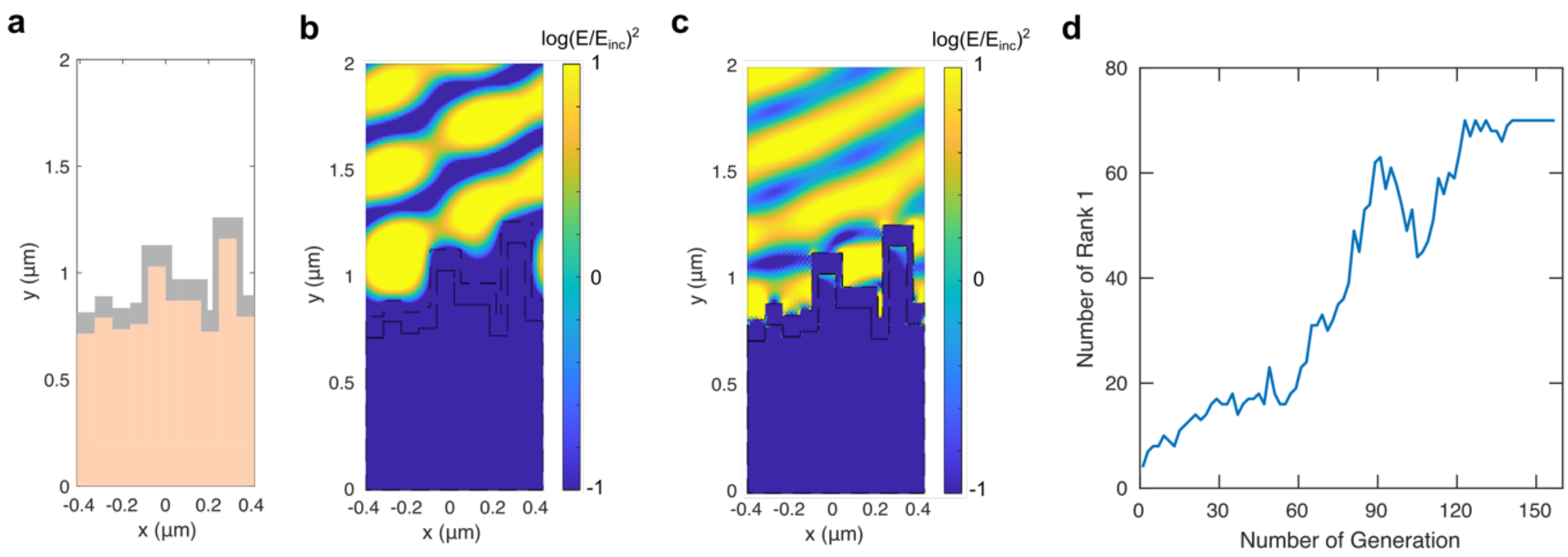

Figure S7. Design optimization of metagratings for polarization-independent beam steering in air. Conditions are set as λ = 633 nm and $\theta_{steering}$ = 50°. (a) Optimized unit cell in air (n = 1.0) achieving steering efficiencies of 0.79 (TE) and 0.78 (TM). Electric field intensity profiles for (b) TE and (c) TM modes. (d) Number of rank 1 over the generation during multi-objective optimization.

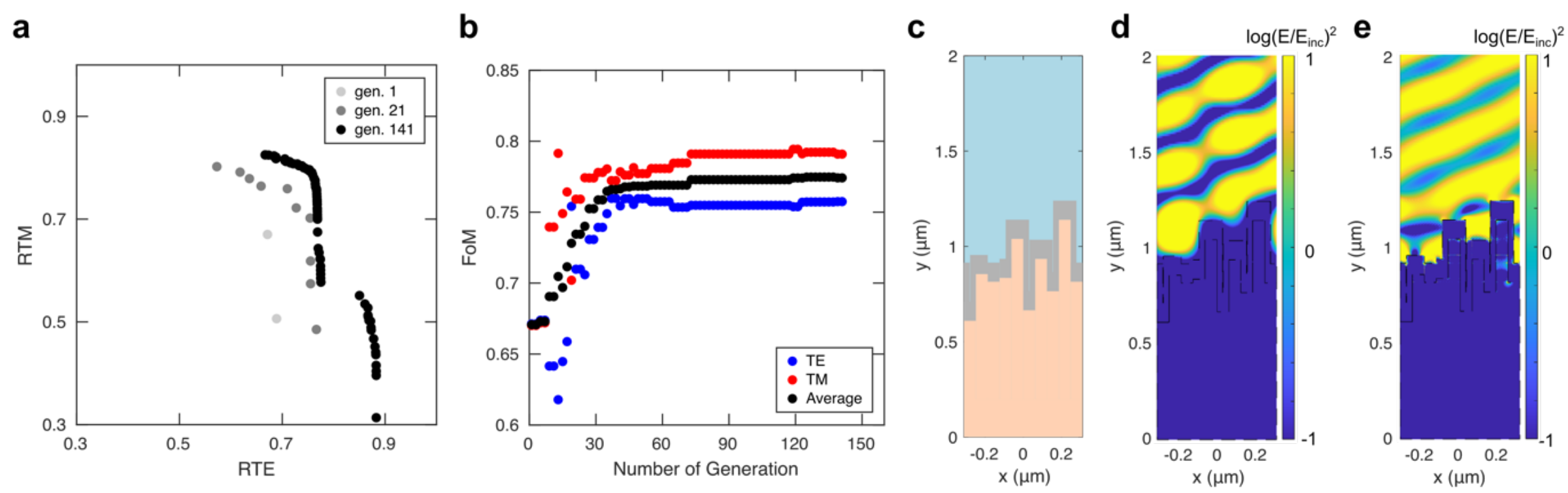

Figure S8. Design optimization of metagratings for polarization-independent beam steering in aqueous environment. Conditions are set as λ = 633 nm and $\theta_{steering}$ = 50°. (a) Evolution of the Pareto front at the 1st, 21st, and 141st generations of the genetic multi-objective optimization. (b) Multi-objective optimization over successive generations, with the figure of merit defined by the knee point that minimizes the distance to the ideal point (TE, TM) = (1, 1). The average steering efficiency shows a gradual increase over generations. (c) Optimized unit cell in water (n = 1.33) achieving steering efficiencies of 0.79 (TE) and 0.76 (TM). Electric field intensity profiles for (d) TE and (e) TM modes.

S7. Mechanism of the Metallic Overlayer and Sidewalls

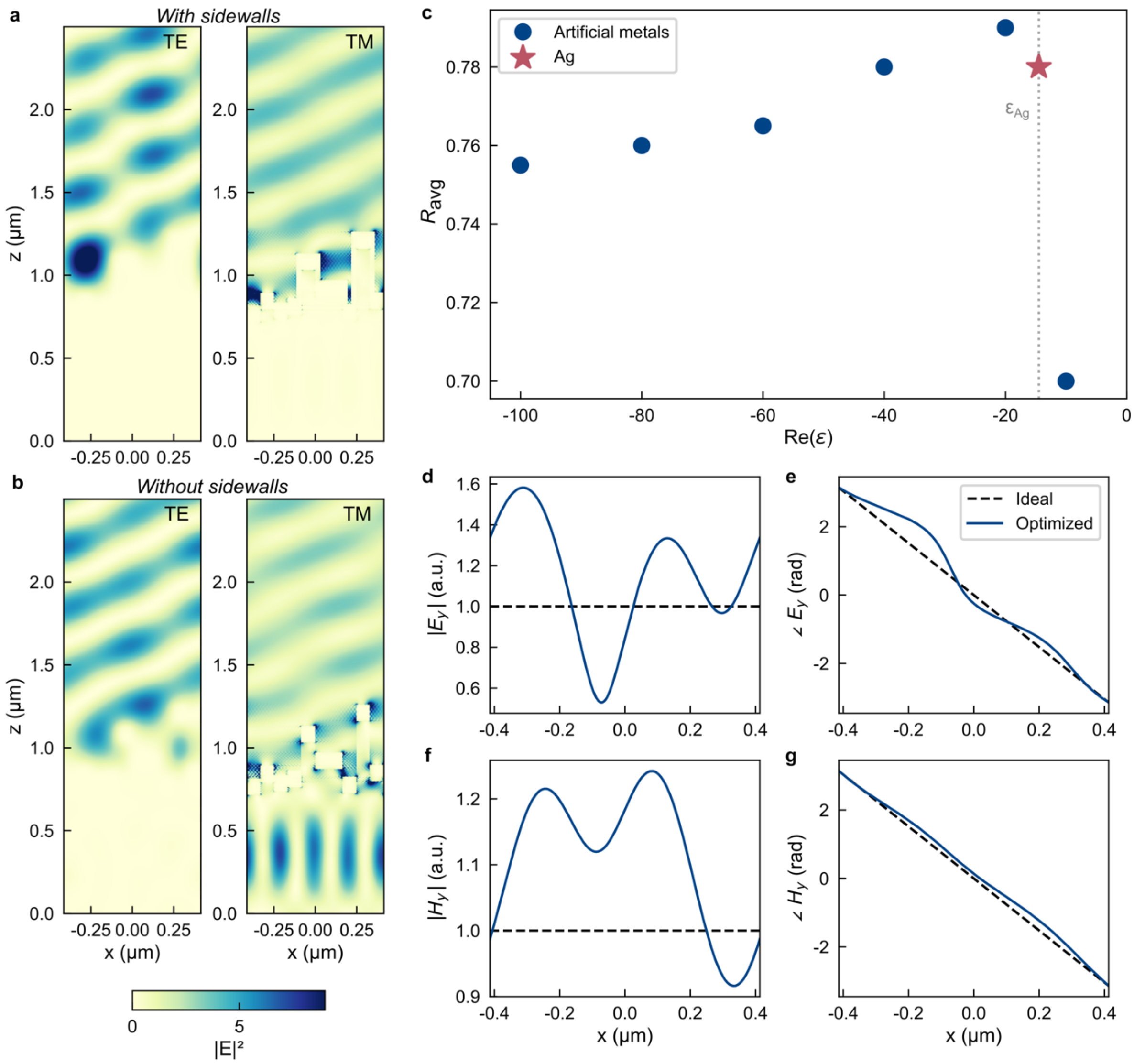

Figure S9: Effect of metallic overlayer and sidewalls on diffraction control. (a) Simulated reflected $|E|^2$ distributions for structures with sidewalls under TE and TM polarization.
(b) Corresponding results without sidewalls for TE and TM, showing pronounced field leakage in the TM mode. (c) Averaged desired order diffraction efficiency (R_{avg}) versus Re(ε); blue circles denote artificial metals, and the red star indicates Silver (Ag), demonstrating weak dependence on permittivity. (d,e) TE-mode amplitude |Ey| and phase ∠Ey, and (f,g) TM-mode amplitude |Hy| and phase ∠Hy, evaluated 200 nm above the topmost metallic layer; dashed lines indicate the ideal profiles.

S8. Comparison of Optical Microsystems

Table S1. Comparison of optical microsystems

Optical microsystem	Actuation modality	Environ-ment	Optical functionality	Design approach	Actuation-optics coupling	Polarization insensitivity	Fabrication	Scalability
Directional scatterer [1,2]	None (static)	Substrate	Directional scattering	Plasmonic/Mie resonance	N/A	No	Bottom-up	Scalable
Magnetic/optical microrobot [3]	Magnetic/Optical	Fluid	Thermo-hydrodynamic actuation	Photothermal effect	Coupled & codependent	Yes	Ultrasonication	Scalable
Photoresponsive soft microrobot [4]	Optical	Fluid	Material deformation	Liquid crystal phase	Coupled & codependent	Yes	Photo-polymerization	Scalable
Diffractive microrobot [5]	Magnetic	Fluid	Beam shaping via deformation	Geometric	Coupled & codependent	N/A	DUV + E-beam lithography	Limited
Optical microrobot [6]	Optical	Fluid	Momentum transfer	Metasurface	Coupled & codependent	No	E-beam lithography	Limited
This work	Magnetic	Fluid	Active beam steering	Multi-objective optimization	Fully decoupled & independent	Yes, polarization-insensitive by design	Nanoimprinting	Wafer-scalable

Table S1—transposed for an alternative view

Optical microsystem	Directional scatterer [1,2]	Magnetic/optical microrobot [3]	Photoresponsive soft microrobot [4]	Diffractive microrobot [5]	Optical microrobot [6]	This work
**Actuation modality**	None (static)	Magnetic/Optical	Optical	Magnetic	Optical	Magnetic
**Environment**	Substrate	Fluid	Fluid	Fluid	Fluid	Fluid
**Optical functionality**	Directional scattering	Thermo-hydrodynamic actuation	Material deformation	Beam shaping via deformation	Optical momentum transfer	Active beam steering
**Design approach**	Plasmonic/Mie resonance	Photothermal effect	Liquid crystal phase	Geometric optics	Metasurface design	Multi-objective optimization
**Actuation-optics coupling**	N/A	Coupled & codependent	Coupled & codependent	Coupled & codependent	Coupled & codependent	Fully decoupled & independent
**Polarization Insensitivity**	No	Yes	Yes	N/A	No	Yes, polarization-insensitive by design
**Fabrication**	Bottom-up	Ultrasonication	Photo-polymerization	DUV + E-beam lithography	E-beam lithography	Nanoimprinting
**Scalability**	Scalable	Scalable	Scalable	Serial-lithography limited	Serial-lithography limited	Wafer-scale compatible

References

1. X. Zhuo, H. K. Yip, Q. Ruan, et al., “Broadside nanoantennas made of single silver nanorods,” *ACS Nano* 12 (2018): 1720–1731.
2. M. L. De Marco, T. Jiang, J. Fang, et al., “Broadband forward light scattering by architectural design of core-shell silicon particles,” *Advanced Functional Materials* 31 (2021): 2100915.
3. M. Sun, S. Yang, J. Jiang, et al., “Multiple magneto-optical microrobotic collectives with selective control in three dimensions under water,” *Small* 20 (2024): e2310769.
4. S. Palagi, A. G. Mark, S. Y. Reigh, et al., “Structured light enables biomimetic swimming and versatile locomotion of photoresponsive soft microrobots,” *Nature Materials* 15 (2016): 647–653.

5. C. L. Smart, T. G. Pearson, Z. Liang, et al., "Magnetically programmed diffractive robotics," *Science* 386 (2024): 1031–1037.
6. D. Andrén, D. G. Baranov, S. Jones, et al., "Microscopic metavehicles powered and steered by embedded optical metasurfaces," *Nature Nanotechnology* 16 (2021): 970–974.
